# Recent Updates on the Role of the MicroRNA-10 Family in Gynecological Malignancies

**DOI:** 10.1155/2022/1544648

**Published:** 2022-12-19

**Authors:** Chunhua Li, Xiangling Zhu, Xinyue Lv, Xintong Han, Yuting Xu, Jinhua Huang, Xiaochun Chen, Zhiying Yu

**Affiliations:** ^1^Department of Gynecology, Shenzhen Second People's Hospital, The First Affiliated Hospital of Shenzhen University Health Science Center, Shenzhen 518035, China; ^2^Institute of Clinical Pharmacology, Anhui Medical University, Key Laboratory of Anti-Inflammatory and Immune Medicine, Ministry of Education, Anhui Collaborative Innovation Center of Anti-Inflammatory and Immune Medicine, Hefei 230023, China

## Abstract

The ever-increasing morbidity associated with gynecological malignancies constantly endangers the physical and psychological health of women. Since a long time, there has been an urgent need for a deeper understanding of the tumorigenesis and the development of gynecological cancer to identify new molecular markers for early diagnosis and metastatic disease prognosis and for the development of therapeutic targets. MicroRNAs are crucial cellular regulators. The microRNA-10 (miR-10) family has been found to play an integral role in the evolution of numerous cancer types. A comprehensive understanding of current studies on miR-10 could provide better insights into future research and clinical applications in related fields. This article reviews the latest research on the role of the miR-10 family in gynecological malignancies and the relevant molecular mechanism, mainly focusing on endometrial, cervical, and ovarian cancers.

## 1. Introduction

Endometrial and cervical cancers are common malignant gynecological tumors associated with an increased incidence. Continuous estrogen stimulation and persistent human papilloma virus (HPV) infection are high-risk factors for endometrial and cervical carcinomas, respectively, but the pathogenesis of these tumors is not well understood [1–4]. Ovarian cancer, although not as common as cervical cancer and endometrial cancer, has the highest mortality rate. Ovarian cancer is often neglected in the early stages, owing to its discreet symptoms and the lack of reliable screening methods. Thus, a deeper understanding of its pathological mechanisms and the identification of prognostic and predictive markers are needed to optimize and personalize the treatment [5, 6].

MicroRNAs (miRNAs) play significant gene-regulatory roles in many cellular functions and are key regulators of the normal and abnormal cell growth and development [7–9]. Accumulating research has shown that the upregulation or downregulation of miRNAs has far-reaching consequences for the tumor development and miRNAs are candidate regulators of oncogenes or tumor suppressors [10]. In endometrial cancer, miRNAs have been found to be aberrantly expressed. Many miRNAs are significantly differentially expressed at different stages of the disease and can be used to differentiate between early and advanced disease [11]. As the morbidity of endometrial cancer is increasing, further research and the development of more broadly applicable and specific screening markers is needed [12]. Functional experiments have also confirmed that some miRNAs have carcinogenic or inhibitory effects in ovarian cancer [13]. In addition, some early studies on cervical cancer have indicated that miRNAs participate in cancer development, growth control, and drug resistance through the regulation of oncogenic or tumor-suppressive targets. miRNAs are also increasingly being identified as cellular regulators with diagnostic, therapeutic, and prognostic values in cervical cancer [3]. There is growing interest in studying the molecular mechanism by which miRNAs affect the occurrence and development of gynecological cancers, to determine potential associations with disease progression, and to assess their efficacy as therapeutic targets [3, 14].

The miR-10 family consists of miR-10a and miR-10b, which have been found to be involved in a variety of physiological and pathological processes [15]. A previous study found that the miR-10 family has several 3′-isomiRs [16]. It has also been documented that the miR-10 family is expressed at different levels in gynecological malignancies [17–19]. This article reviews the literature on the functions of miR-10a and miR-10b in endometrial, cervical, and ovarian cancers, with the objective of highlighting the important role of the miR-10 family in gynecological malignancies and proposing future research directions.

## 2. The Role of miR-10a in Reproductive System-Related Cancers

### 2.1. Endometrial Cancer

**Figure 1:**
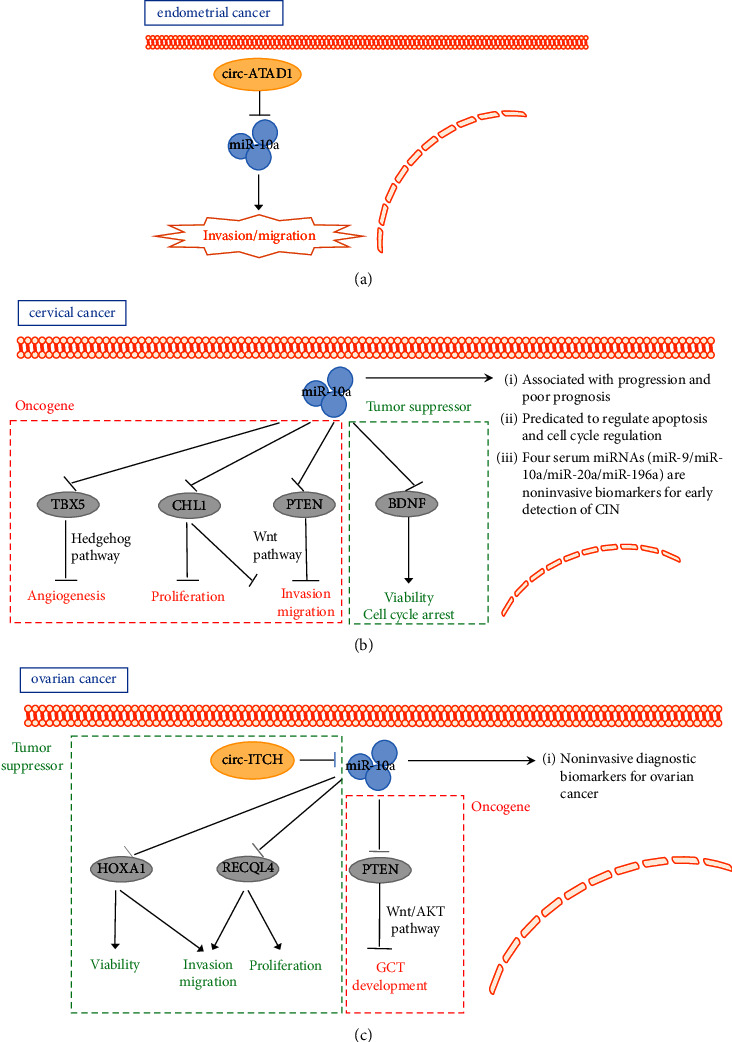
The role of miR-10a in female reproductive tumors. (a) miR-10a is regulated by circ-ATAD1 to inhibit endometrial cancer cell invasion and migration. (b) miR-10a promotes cervical cancer cell angiogenesis, proliferation, invasion, and migration by targeting TBX5, CHL1, and PTEN; miR-10a decreases cervical cancer cell viability and inhibits cell cycle arrest by targeting BDNF. (c) miR-10a is regulated by circ-ITCH in ovarian cancer; miR-10a decreases ovarian cancer cell viability and inhibits invasion, migration, and proliferation by targeting HOXA1 and RECQL4. miR-10a promotes the GCT development by targeting PTEN via Akt and Wnt pathways.

Yang et al. [20] revealed that circ-ATAD1 expression is elevated in endometrial cancer but miR-10a expression decreased. In endometrial carcinoma cells, the overexpression of circ-ATAD1 was found to inhibit the miR-10a expression. Further, circ-ATAD1 and miR-10a are negatively associated in endometrial tumor tissues. MiR-10a is also regulated by oncogenic circ-ATAD1 to inhibit the cell invasion and migration (Figure 1(a)).

### 2.2. Cervical Cancer

Zhang et al. [21] found that miR-10a is highly expressed in cervical tumors. *In vivo*, injecting nude mice with cancer associated fibroblast-extracellular vesicles (CAF-EVs) and then treating them with a miR-10a-5p inhibitor contributed to a decrease in the tumor bulk and growth of peripheral vessels. In addition, the study showed that TBX5 is a target gene of miR-10a-5p. Moreover, miR-10a-5p derived from the CAF-EVs was found to be involved in tumor angiogenesis, via the downregulation of TBX5-activated Hedgehog signaling. Another study revealed the aberrant regulation of miR-10a expression in cervical cancer. However, in a short term, miR-10a may have no or little effect. Meanwhile, miR-10a can target CHL1 and reduce its expression levels to promote long term tumor growth and metastasis [22]. Zeng and Li [23] found that miR-10a is significantly upregulated in patients with advanced cervical tumors. In addition, miR-10a may affect the migration and invasion of cervical carcinoma cells by repressing an essential tumor suppressor, PTEN (phosphatase and tensin homolog).

Safari et al. [24] found that miR-10a expression is higher in cancerous tissues than in paracervical tissues. This study analyzed the correlation between miR-10a expression and cervical cancer stage, grade, and tumor growth. miR-10a was identified as an oncogene and found to be related to poor prognosis in cervical cancer. Cheung et al.[25] also demonstrated aberrant miR-10a expression via the surveillance analysis of cervical intraepithelial neoplasia (CIN) and normal cervical epithelial samples. This analysis suggested that miR-10a may have a role in distinguishing normal cervical epithelium from high-grade cervical precancerous lesions and in regulating apoptosis and growth. Another cohort study revealed that miR-9, miR-10a, miR-20a, and miR-196a are expressed at elevated levels in patients with CIN. This feature is important for the identification of individuals with CIN. These aberrantly expressed miRNAs may be precancerous biomarkers of cervical cancer and could be used for early detection [26]. However, the specific role of miR-10a in cervical cancer is not conclusive. Zhai et al. [27] revealed that miR-10a-5p expression is inhibited in cervical cancer. miR-10a-5p can also inhibit the growth of cervical carcinoma cells while regulating and controlling the expression of the BDNF (brain derived neurotrophic factor) gene (Figure 1(b)).

### 2.3. Ovarian Cancer

Liu et al. [28] suggested that miR-10A-5p expression is suppressed in ovarian tumors, as it targets HOXA1 to inhibit ovarian cancer growth and metastasis. Another study demonstrated that miR-10a-5p targets RECQL4 in ovarian cancer cells to inhibit cell proliferation and viability [29]. In addition, Luo et al. [30] performed a series of experiments to show that circ-ITCH inhibits miR-10a expression in ovarian carcinomas. circ-ITCH unidirectionally regulates miR-10a expression and inhibits tumor cell growth. In addition, a case-control study showed that several miRNAs are differentially expressed intracellularly, extracellularly, and in urine from patients with ovarian cancer. Among them, the miR-10a expression tends to be mildly downregulated in the urine of ovarian cancer patients. In this study, it was suggested that the changes in the miRNA expression in intracellular and extracellular compartments and in urinary biological fluids indicate that they may be potential biomarkers for liquid biopsies [31]. Further, Wang et al. [32] experimentally found that miR-10a is abnormally expressed in plasma from ovarian cancer patients and thus could be a noninvasive molecular biomarker for ovarian cancers. Benson et al. [33] showed that plasma concentrations of miRNAs, including miR-10a-5p, are changed significantly in patients with platinum-resistant, recurrent ovarian cancer after the chemotherapy. Tu et al. [34] demonstrated the effect of miR-10a on granulosa cell tumors (GCTs) by using *in vivo* and *in vitro* GCT models. miR-10a was found to promote the GCT development by targeting the PTEN and Wnt pathways (Figure 1(c)).

## 3. The Role of miR-10b in Reproductive System-Related Cancer

### 3.1. Endometrial Cancer

**Figure 2:**
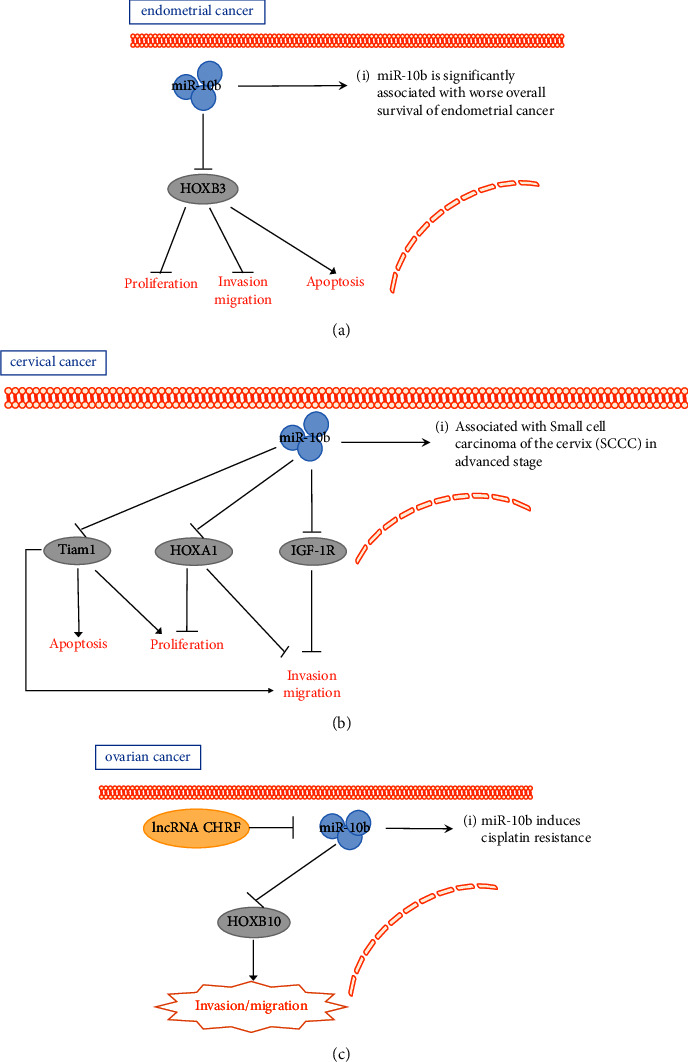
The role of miR-10b in female reproductive tumors. (a) miR-10b promotes endometrial cancer cell apoptosis and inhibits cell proliferation, migration, and invasion by targeting HOXB3. (b) miR-10b promotes cervical cancer cell apoptosis and inhibits cell proliferation, migration, and invasion by targeting Tiam1, HOXA1, and IGF-1R. (c) miR-10b is repressed by lncRNA CHRF in ovarian cancer; miR-10b promotes ovarian cancer cell migration and invasion by targeting HOXB10 in ovarian cancer.

Two studies have suggested that miR-10b expression levels are significantly elevated in endometrial cancer tissues. In a study by Chen et al. [35], after miR-10b silencing, HOXB3 protein levels increased in the endometrial cancer cell line KLE. Furthermore, the HOXB3 overexpression was found to promote apoptosis but inhibit the proliferation, migration, and invasion of KLE cells. miR-10b may regulate the growth and metastasis of endometrial tumor cells by adjusting and controlling the expression of HOXB3 [36]. Another study identified differential miRNA expression in endometrioid endometrial carcinoma tissue and plasma using next-generation sequencing, with miR-10b expression being downregulated [37]. Hiroki et al. [38] demonstrated that miR-10b and miR-10b^*∗*^ (passenger chain at the miR-10b locus) expressions are downregulated in endometrial serous adenocarcinoma (ESC) and is also a potential tumor prognostic marker. Experiments have also shown that reduced miR-10b^*∗*^ expression is related to the vascular invasion in endometrial cancer (Figure 2(a)).

### 3.2. Cervical Cancer

Zou et al. [39] discovered that miR-10b is expressed at a low level in cervical cancer. Functional assays confirmed that miR-10b may be a tumor suppressor gene that regulates the growth and migration of cervical cancer cells by directly targeting HOXA1. Yu et al. [40] experimentally showed that in cervical cancer, low expression of miR-10b is associated with tumor volume, vascular infiltration, and human papilloma virus type 16 (HPV-16, the most prevalent genotype of high-risk human papillomavirus detected in cervical cancer) infection [41, 42]. MiR-10b exerts a tumor-suppressive effect in cervical cancer by targeting the oncogenic factor Tiam1. Moreover, the miR-10b expression may be downregulated by the HPV-methylated TFAP2A-binding element. Hou et al. [43] also showed that the miR-10b is expressed at low levels in cervical cancer tissues. Further, miR-10b was found to exert inhibitory effects in cervical tumors by targeting IGF-1R. Chen et al. [44] investigated the association between miRNA single nucleotide polymorphisms (SNPs) and CIN and cervical cancer, including rs107822 in miR-219a, rs10877887 in let-7i, rs2292832 in miR-149, rs353293 in miR-143, rs3746444 in miR-499, rs3803808 in miR-132, rs4078756 in miR-10b, rs629367 in let-7a, and rs7372209 in miR-26a. No association was found between these SNPs and CIN. This study suggested that miRNA gene polymorphisms may affect miRNA expression, regulate PI3K/Akt signaling, and play a significant role in cancer susceptibility. Huang et al. [45] showed the significant downregulation of has-miR-10b expression in advanced small squamous cell carcinoma (SCCC, an aggressive, rare form of cervical cancer, accounting for fewer than 3% of all cervical cancers), but the correlation between lymph node metastasis and survival in SCCC is unclear. Sommerova et al. [46] indicated that miR-10b-5p is expressed at low levels in dysplastic cervical tissue, in contrast to the pattern observed for the miR-10a-5p expression. In addition, miR-10a-5p is an oncogenic miRNA, whereas miR-10b-5p can be characterized as an miRNA with tumor suppressive effects in cervical tissue (Figure 2(b)).

### 3.3. Ovarian Cancer

Tan et al. [47] observed that miR-10b is regulated by CHRF (cardiac hypertrophy-related factor) to induce epithelial-mesenchymal transition and activate the STAT3 signaling pathway and mediate cisplatin resistance. Moreover, miR-10b plays a key role in the treatment of cisplatin-resistant ovarian cancer. Nakayama et al. [48] showed that excessive miR-10b expression can target HOXD10 to affect the migration and invasive ability of ovarian cancer cell lines. The expression of the premetastatic gene products MMP14 and RHOC may also be influenced by miR-10b (Figure 2(c)).

## 4. Discussion

The first miRNA, Let-7, was identified in *Caenorhabditis elegans* in 1993 [49]. More than 20 years since the discovery of the first miRNA, considerable progress has been made in the study of the roles of these small noncoding RNAs [50, 51]. The refinement of cell function experiments and target analysis has led to an increasing recognition of the role of miRNAs in the tumor development [20, 31, 40]. In a recent article, Weinberg[52] proposed that there are eight hallmarks of tumors required for the activation of tumor growth and progression, including the acquired maintenance of proliferative signaling, evasion of growth inhibitors, resistance to cell death, accomplishment of replicative immortality, induction/entry into the vascular system, activation of invasion and metastasis, reprogramming of cellular metabolism, and ability to evade immune destruction, along with two enabling features, including the promotion of tumor inflammation and genomic instability, and mutagenesis. The eight features proposed by Weinberg provided more directions for future scholars to study the effect of the miR-10 family on tumors. The current review focused on research progress on the role of the miR-10 family of miRNAs in gynecological malignancies. There are many studies on the role of the miR-10 family in reproductive system-related tumors. At present, there has not been a comprehensive review of research progress on the miR-10 family in reproductive system-related tumors. Therefore, this article provides a novel review of this subject. An increasing number of studies has indicated that miR-10a expression is dysregulated in tumors associated with the reproductive system and it exerts oncogenic or suppressive effects by regulating different targets. Nevertheless, some studies have concluded the opposite results [27, 28, 34]. More solid *in vivo* experiments are needed to better understand the specific role of the miR-10 family in different tumors.

Since the effect of miRNAs in cancer continues to be uncovered, the application of miRNAs to cancer diagnosis and therapy is becoming more attractive [53]. Current research tends to be increasingly focused on the mechanisms of miRNA interactions with regulatory genes, oncogenes, tumor suppressors, and also individual miRNA target genes [54]. Aberrant miRNA expression profiles have been of interest to many researchers and are being used to identify disease states of cancers [55]. miRNA expression profiles reinforce the precise classification of multiple types of cancer [56]. Research has increasingly focused on the transfer of tumor-associated miRNA isoforms and exosome-mediated miRNAs to other cells and the association between these miRNAs and cancer. Increasingly, aberrant miRNA expression has been identified in the serum. The curiosity in miRNAs is rapidly growing, as a potential biomarker of interest in the field [26, 57]. The increasing number of experiments demonstrating the abnormal expression of miRNAs in blood specimens from tumor patients implies that miRNAs may be a potential noninvasive biomarker for the diagnosis and prognosis of cancer [39]. Interestingly, miR-10 is gaining interest as a promising noninvasive tumor marker for the early identification of tumors and prediction of chemotherapy responses with germline-associated tumors [33]. The presence or absence of lymph node metastasis is a principal factor in the diagnosis, treatment, and prognosis of cancer. Therefore, the development of tumor markers for the detection of lymph node metastasis is crucial. It has been shown that miR-10b is dysregulated in cervical cancer patients with lymph node metastasis and could be a potential biomarker [23]. In addition, there are also some advances in miRNA-based tumor therapies. Many studies have demonstrated through cellular experiments that miRNAs are dysregulated in cancer and the dysregulated miRNAs exert oncogenic or tumor-suppressive effects. Further, miRNA mimics and molecules targeting miRNAs show potential for the development of new diagnostic and therapeutic approaches [58]. A mimetic tumor suppressor miR-34 has completed phase I clinical trials (Clinical trial No. NCT01829971) for the treatment of cancer, whereas an anti-miRNA, miR-122, targeting miR-122 has reached phase II clinical trials (Clinical trial No. NCT01200420) for the treatment of hepatitis, suggesting the prospect of miRNA-targeted therapies being used for the clinical treatment [59].

It has been shown that miRNAs can be used in combination with immunotherapy, radiotherapy, and chemotherapy for the cancer treatment. This is certainly a new approach when compared to miRNA-based therapies, the use of miRNA mimics, and alternative treatments. Moreover, the use of advanced therapeutic approaches, such as complementary miRNAs, offer potential clinical benefits for cancer patients. Current clinical trials applying miRNAs for the treatment of cancer have shown to be successful [60]. The role of miR-10 in reproductive system-related tumorigenesis and progression needs to be understood. Such studies may be complex owing to the presence of multiple miR-10 family members, which could lead to gene redundancy. In addition, knockout and transgenic models are critical for exploring the functional importance of miR-10 in tumors. Although several studies have shown that miR-10 can target different genes, the regulatory networks in which miR-10 is involved are poorly understood. Identifying the upstream and downstream regulatory pathways of miR-10 and obtaining a comprehensive picture of miR-10 targets in different cell types should thus be a future direction. Research on miR-10 has also made great progress for diagnostic and therapeutic purposes in germline-associated tumors; however, miRNAs, as cancer biomarkers and therapeutic tools, have not yet been translated into the clinic, and there is still much work to be performed. Understanding the molecular pathways that regulate miR-10 in the development of reproductive system-associated tumors could provide insights into its clinical application to gynecological cancers and to a wider range of diseases. It may be too early to discuss the use of miR-10 as a future targeted therapy; however, the increasing trend in the number of new discoveries over the past decade is certainly encouraging and promising.

At present, many studies have shown that the expression of miRNAs is closely related to many complex human diseases. It is difficult to form a comprehensive understanding of the relationship between the huge miRNA system and complex diseases. In recent years, owing to the establishment of a large number of noncoding RNAs and disease association databases, computational models to predict the potential association between miRNA and disease, using computational methods, have achieved great success. Li et al. introduced the origins and functions, a database, computational model, and other research progress on miRNAs and complex diseases through a series of reviews. With the update of the database and the accumulation of experimental data, the relationship between miRNA and complex diseases has become increasingly clear [61, 62]. Through large-scale inferences of miRNA associations with disease, such computational models, can yield the most advanced predictive results; this can effectively reduce requirements for disease screening in biological experiments, significantly improve the efficiency for biological detection, and greatly reduce the time and cost of biological experiments. These methods have the potential to be used to screen miRNA sets related to malignant tumors of the reproductive tract in the future. At the same time, with the increasing size of data sets and database updates, the development of powerful and special computational models is also worth exploring [61–65].

## Data Availability

This is a review article, and all cited data can be found on Pubmed.
